# Late Repair of Aortic Pseudoaneurysm After Trans-Femoral Aortic Valve Implantation

**DOI:** 10.3390/jcm15114050

**Published:** 2026-05-24

**Authors:** Juan Esteban de Villarreal-Soto, Juan Francisco Oteo-Domínguez, Alberto Forteza-Gil

**Affiliations:** 1 Cardiac Surgery Department, Hospital Universitario Puerta de Hierro Majadahonda, 28222 Majadahonda, Spain; 2 Cardiology Department, Hospital Universitario Puerta de Hierro Majadahonda, 28222 Majadahonda, Spain; 3 Cardiac Surgery Department, Hospital Universitario Quironsalud, 28223 Pozuelo, Spain

**Keywords:** aortic annulus rupture, transcatheter aortic valve implantation, computed tomography

## Abstract

We present the case of a patient who underwent transcatheter aortic valve implantation and subsequently developed an aortic pseudoaneurysm. During follow-up, the patient appeared clinically and radiologically stable, although the imaging modalities employed were not adequate to fully assess the lesion. Ten months later, a thoracoabdominal computed tomography scan performed for an unrelated reason revealed significant pseudoaneurysm growth, and the patient subsequently underwent complex aortic root surgery. **Central Message:** Different degrees of iatrogenic aortic annulus rupture after TAVI lead to different therapeutic approaches; once the acute phase is over it requires close follow-up with appropriate imaging techniques.

## 1. Introduction

Transcatheter aortic valve implantation (TAVI) has transformed the management of severe aortic stenosis (AS) over the past two decades, evolving from a rescue strategy in inoperable patients to an established alternative for individuals across virtually all surgical risk categories. The landmark PARTNER trial series demonstrated non-inferiority of TAVI compared to surgical aortic valve replacement (SAVR) with regard to mortality, stroke and rehospitalization at seven-year follow-up [1]. As procedural volumes have grown exponentially worldwide, so too has recognition of previously underappreciated complications. Among these, aortic annulus rupture (AAR) after TAVI is a rare but potentially catastrophic complication, with a reported incidence ranging from 0.6% to 1.1% in larger registry studies, though its true frequency is likely underestimated [2,3]. Some series report that minor AARs, presenting as small tears, are considerably more common than registries suggest, as the sealing skirt of the TAVI frame effectively tamponades the defect and prevents extravasation detectable on periprocedural imaging [3]. The underlying mechanism of AAR relates to the radial forces generated during balloon inflation or self-expansion of the prosthetic frame against a non-compliant, heavily calcified annulus. When the circumferential stress exceeds tissue tolerance, a tear can propagate through the annular tissue into surrounding structures, including the aortic root, left ventricular outflow tract (LVOT), or interventricular septum. The extent and direction of the tear determine the clinical spectrum, ranging from an asymptomatic contained hematoma to a life-threatening pseudoaneurysm or frank cardiac tamponade. Different authors have identified prosthesis oversizing greater than 20%, heavy calcification of the LVOT, and the use of balloon-expandable valves (BEV) as the principal procedural risk factors for AAR [2,3]. Additional patient-level factors that have been implicated include a small aortic root with a narrow sinotubular junction, pre-existing aortic root pathology, porcelain aorta, and a high ratio of annular calcium volume to annular area as measured on pre-procedural multiplanar, ECG-gated computed tomography (CT) scan. Patient age and the degree of subannular calcification extending into the membranous septum have also been correlated with risk in some cohorts [4]. An AAR should be suspected when a patient fails to achieve hemodynamic stability following valve deployment, or when new pericardial effusion, hypotension refractory to vasopressors, or unexplained thoracic pain emerges in the periprocedural period. Diagnostic confirmation relies on a combination of aortography performed during the index procedure, transesophageal echocardiogram (TEE), transthoracic echocardiography (TTE), and most definitively CT, with the last being the only modality capable of providing reliable volumetric assessment of contained lesions such as pseudoaneurysms and annular hematomas [2].

We present the evolution of a patient who underwent TAVI with BEV who developed an aortic pseudoaneurysm, apparently stable due to not using the appropriate diagnostic technique. Ten months later after performing a thoracoabdominal CT scan for another reason, significant growth was observed. The patient underwent complex aortic root surgery.

## 2. History of Presentation

This is a patient evaluated by the Pulmonology Department as an outpatient for follow-up of a pulmonary nodule; he remained asymptomatic at that time. However, thoracoabdominal CT demonstrated significant growth of an aortic pseudoaneurysm. Consequently, the patient was referred to our center for further management. Upon admission, the patient presented in good general condition, with normal mental status and level of consciousness. He was eupneic at rest, normohydrated, and well-perfused. A regular heart rate with systolic and diastolic murmurs was noted at the aortic focus. Breath sounds were normal, and there was no peripheral edema or evidence of deep vein thrombosis.

## 3. Past Medical History

84-year-old patient who underwent TAVI with a BEV (prosthesis-to-annulus area oversizing of 11.3%) for severe AS 6 months ago. Pre-procedural CT scan showed moderate-to-severe aortic valve calcification, including eccentric calcification involving the left coronary sinus and left ventricular outflow tract (Figure 1A,B). The patient presented a complicated postoperative period with a cerebrovascular accident that was managed conservatively in the absence of obstructive lesions. On echocardiographic follow-up, he presented mild residual aortic insufficiency (AI) with hematoma contained in the annulus under the left coronary sinus.

## 4. Differential Diagnosis

The different acute mechanical complications post-TAVI are valve migration, occlusion of the coronary ostia, aortic dissection, annular rupture and myocardial rupture. All of these complications, except the latter, can be evidenced with fluoroscopy during the procedure, which would require a TTE/TEE or a CT scan. Myocardial rupture can occur in the interventricular septum or in the free wall of both ventricles. Myocardial and annular rupture clinical presentation can vary from asymptomatic pseudoaneurysms to cardiac tamponade and shock. With the proper diagnostic tools, the differential diagnosis between these complications can be assessed. Aortic dissection and coronary ostia occlusion present with hemodynamic collapse; in the case of coronary occlusion, electrocardiographic abnormalities are evidenced.

## 5. Investigations

A chest CT was performed, and the patient was diagnosed with AAR below the trunk of the left coronary artery with an image of a 2 × 1.5 cm pseudoaneurysm (Figure 2A–C), stable in three subsequent controls using TTE and TEE with apparently adequate visualization (although visualization may have been partially limited by prosthetic shadowing from the TAVI frame and/or patient’s echocardiographic window, which likely contributed to the underestimation of pseudoaneurysm growth) (Figure 3A–D). The patient was discharged with no clinical symptoms. Repeat chest CT was not performed during the index hospitalization or at early follow-up, as serial echocardiographic studies suggested lesion stability. A thoracoabdominal CT scan was performed to monitor pulmonary nodules during patient follow-up, where pseudoaneurysm growth of 2 to 8 cm was observed. At the patient’s arrival TTE was repeated observing the biological aortic prosthesis with dehiscence causing moderate aortic insufficiency, which runs posteriorly through the aorto-ventricular junction. In addition, the already known pseudoaneurysm was observed in the aortic root, towards pulmonary artery measuring 2.2 × 2.3 cm, without fistulas. A new ECG-gated CT scan was repeated at our center confirming pseudoaneurysm growth (Figure 2D–F).

## 6. Management

Due to pseudoaneurysm growth, this case was discussed in a medical-surgical session to be operated on by Cardiac Surgery for the implantation of a valved tube Bentall procedure. The surgical procedure was performed through median sternotomy and cardio-pulmonary bypass (CPB). Aortic clamping was performed with myocardial protection using antegrade cardioplegia. A transverse aortotomy was performed and the TAVI was removed, revealing the described pseudoaneurysm dependent on the left coronary sinus due to AAR. Due to the extensive destruction of the annulus, replacement of the root, ascending aorta, and aortic valve using a valved tube was required. Upon leaving CPB, the patient required support with an intra-aortic balloon pump (IABP). As a postoperative complication in the ICU, the patient presented atrial fibrillation, which was reversed with pharmacological management. The IABP was removed on the first postoperative day and the patient was discharged from the ICU on the second postoperative day. In the Cardiac Surgery ward, medication adjustment and management by Infectious Diseases and Urology are carried out due to right epididymitis with reactive hydrocele. The patient was discharged home on the 14th day without incidents. Figure 4 summarizes the timeline of clinical course, imaging findings and management.

## 7. Discussion

In our experience with the TAVI program from 2008 to present day, and that was reported in different publications [2,3,4,5,6], we have observed different degrees of AAR. This case, combined with the existing literature, reinforces the need for a structured prognostic classification of AAR to guide clinical decision-making and define appropriate imaging surveillance strategies. We propose organizing AAR into three broad phenotypes according to severity and anatomical containment (Table 1 summarizes these three phenotypes).

At the most severe end of the spectrum lies the uncontained or free AAR, an immediately life-threatening event in which the annular tear communicates openly with the pericardial space or mediastinum, producing cardiac tamponade, circulatory collapse, and death within minutes if not surgically corrected [2,3,7]. Mortality in this group, even with prompt surgical rescue, remains exceedingly high in elderly and frail patients. In those who can be stabilized long enough to reach the operating room, the surgical strategy involves removal of the TAVI device, aortic valve replacement, and reconstruction of the annulus and surrounding tissue either with a pericardial patch or, when annular destruction is extensive, with implantation of a valved conduit as a modified Bentall procedure’ the approach was required in the present case [7].

At the opposite, more favorable end of the spectrum are small, contained annular tears that form fistulous communications into adjacent right-sided cardiac chambers, most commonly the right ventricle or right atrium. These aorto-cardiac fistulas typically produce a left-to-right shunt, generate a continuous murmur audible at the left sternal border, and behave relatively benignly over time. Because they are small and flow-limited, they are generally well visualized on TTE and TEE, which reliably identifies the shunt jet, characterizes its direction and size, and monitors for progressive enlargement [5,6]. Most of these fistulas remain stable without intervention, though close surveillance is warranted given that progressive shunt enlargement can eventually lead to right ventricular volume overload and heart failure.

Between these two extremes lies an intermediate phenotype that is arguably the most clinically treacherous: the contained AAR, which encompasses annular hematomas, pseudoaneurysms, and limited perforations sealed by surrounding periaortic tissue. This intermediate group is the focus of the present report. These lesions are notoriously difficult to characterize by echocardiography alone. TEE offers superior resolution compared to TTE and should be the initial modality of choice in hemodynamically unstable patients, but its two-dimensional cross-sectional perspective imposes important limitations when assessing the three-dimensional extent and volume of a periaortic pseudoaneurysm. As demonstrated in our case, four serial echocardiographic studies, both TTE and TEE interpreted as “stable” were in fact failing to detect a pseudoaneurysm that had grown from 2 cm to 8 cm over a ten-month period. This finding is consistent with prior reports by Himbert et al. [5] and Cobiella et al. [6], who similarly documented contained aortic ruptures in the TAVI setting that were underestimated by ultrasound-based imaging.

The role of CT in the surveillance of intermediate AAR phenotypes deserves particular emphasis. Multiplanar, ECG-gated CT scan provides unrivaled spatial resolution and the ability to reconstruct the aortic root and periaortic structures in three dimensions, making it uniquely suited to measure pseudoaneurysm volume, delineate the neck of the communication with the aortic lumen, assess proximity to coronary ostia, and detect progressive enlargement over sequential imaging. In our center’s experience, the growth from 2 cm to 8 cm was identified only when a CT was obtained incidentally for an unrelated indication, a circumstance that should not be the standard of care for these patients. In this case, a peri-annular hematoma was already documented six months before the incidental CT finding, representing a missed opportunity for earlier cross-sectional imaging. We therefore propose that a dedicated ECG-gated CT of the aortic root should be mandatory at 3 months following any TAVI procedure in which a contained annular injury, including hematoma, minor perforation, or pseudoaneurysm, is identified, regardless of echocardiographic stability, with repeat imaging at 6 and 12 months if the lesion remains present and stable. Criteria for surgical intervention should include size exceeding 3 cm, growth between sequential studies, evidence of prosthetic dehiscence, or the development of symptoms attributable to the pseudoaneurysm such as chest pain, dyspnea, or new-onset aortic regurgitation.

With respect to the pre-procedural prevention of AAR, the importance of accurate annular sizing cannot be overstated. High-resolution CT-based annular area measurement has replaced echocardiographic sizing as the standard of care at most experienced TAVI centers, because it more accurately reflects the elliptical geometry of the native aortic annulus and allows precise calculation of the prosthesis-to-annulus area ratio. Avoiding prosthesis oversizing beyond 10–15% in patients with heavy LVOT calcification, even at the cost of accepting some residual paravalvular regurgitation, is a pragmatic strategy that has been adopted by several groups to mitigate rupture risk. The use of a deliberate undersizing strategy, combined with a sizing algorithm that accounts for total calcium volume and its circumferential distribution, may further reduce the incidence of this complication as TAVI is applied to progressively younger and lower-risk patients in whom long-term structural consequences of annular injury carry greater weight.

From a surgical perspective, the Bentall procedure performed in this case illustrates several important technical considerations unique to the TAVI-to-SAVR conversion setting. Extensive annular destruction, as encountered in this patient, necessitates root replacement rather than simple valve exchange, adding operative complexity and cardiopulmonary bypass time in a population that is by definition intermediate-to-high-risk. In our case, the patient required intra-aortic balloon pump support on coming off CPB, consistent with the degree of myocardial stress associated with this procedure. Nevertheless, the favorable outcome of ICU discharge on postoperative day 2, hospital discharge on day 14, and full return to daily activities, demonstrates that surgical rescue is achievable with careful multidisciplinary planning and operative technique.

Looking ahead, several areas warrant further investigation. First, the natural history of intermediate-grade AAR lesions managed conservatively remains poorly characterized. Prospective registries with mandatory CT follow-up at standardized time points are needed to define growth rates, predictors of expansion, and threshold sizes at which elective surgical or transcatheter repair is justified. Second, the feasibility of transcatheter approaches to pseudoaneurysm exclusion in patients who are deemed prohibitive surgical risks deserves evaluation. In patients deemed prohibitive surgical candidates due to advanced age, frailty, or prior sternotomy, percutaneous exclusion of the pseudoaneurysm represents a valuable alternative. Salido et al. and Katada et al. have reported successful transcatheter closure using vascular plugs and coils in this setting, achieving adequate pseudoaneurysm exclusion without the need for cardiopulmonary bypass. Although experience remains limited to small series and individual case reports, these techniques merit consideration in a multidisciplinary heart team discussion when open surgical repair carries prohibitive risk [8,9]. Third, the introduction of next-generation TAVI platforms with improved conformability, repositionability, and reduced radial force profiles may alter the incidence and pattern of AAR, and comparative data across device generations will be important to accumulate systematically. Finally, consensus guidelines from the major cardiovascular societies on the optimal imaging protocol for post-TAVI surveillance of annular complications are currently lacking; the present case adds to the body of evidence supporting the inclusion of CT-based follow-up in any patient with a documented or suspected annular injury at the time of TAVI.

## 8. Follow-Up

During follow-up in Cardiac Surgery consultations, a TTE was performed, showing normally functioning aortic prosthesis, with no other signs of complications. The patient remains asymptomatic and has returned to his daily activities.

## 9. Conclusions

This case illustrates a critical and underappreciated clinical problem: the silent progression of a TAVI-related aortic pseudoaneurysm under conventional echocardiographic surveillance. TTE and TEE, while valuable for the detection of fistulous connections and real-time hemodynamic assessment, are fundamentally limited in their ability to characterize the three-dimensional volume and growth trajectory of periaortic contained lesions. CT remains the only imaging modality capable of providing this information reliably, and its integration into structured follow-up protocols for patients with documented annular injury is not optional, it is essential. The clinical spectrum of iatrogenic AAR after TAVI ranges from immediately life-threatening free rupture requiring emergent surgical rescue to indolent pseudoaneurysms that grow silently over months to years, and from aorto-cardiac fistulas that may remain stable indefinitely to contained hematomas that evolve unpredictably. Each phenotype demands a distinct management strategy, and no single imaging modality covers the full diagnostic range. A multimodal surveillance approach, anchored by ECG-gated CT at defined intervals, should be standardized in centers performing TAVI.

## 10. Learning Objectives

1. In patients with mild aortic annulus rupture after TAVI implantation that causes fistulae to right cavities, follow-up with TEE or TTE will illustrate its evolution.
2. Perform a computer tomography scan when aortic hematoma, pseudoaneurysms or contained rupture are suspected, to obtain an adequate three-dimensional assessment of its size and growth.
3. Aortic annulus rupture management varies depending on its degree, ranging from close monitoring to emergent surgery.

## Figures and Tables

**Figure 1:**
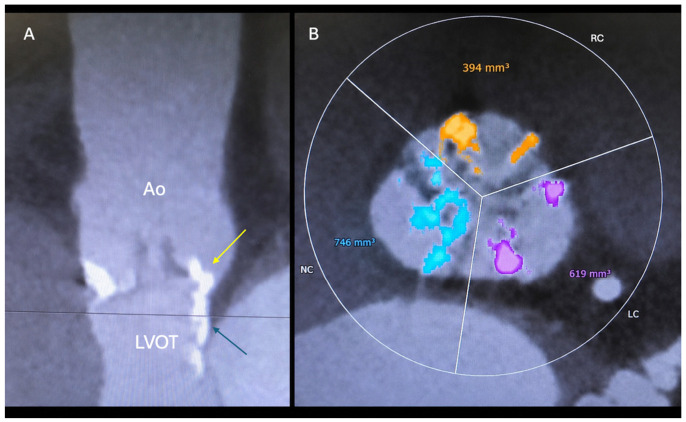
Pre-TAVI cardiac CT: (**A**) MPR reconstruction of the aorta and left ventricular outflow tract showing moderate-to-severe aortic valve calcification, including eccentric calcification involving the left coronary sinus and left ventricular outflow tract. (**B**) Aortic valve calcium cuantification. Ao: Aorta. Blue arrow: Calcium in the LVOT. LC: Left coronary sinus. LVOT: Left ventricle outflow tract. NC: Non-coronary sinus. RC: Right coronary sinus. Yellow arrow: Excentric calcium in the left coronary sinus.

**Figure 2:**
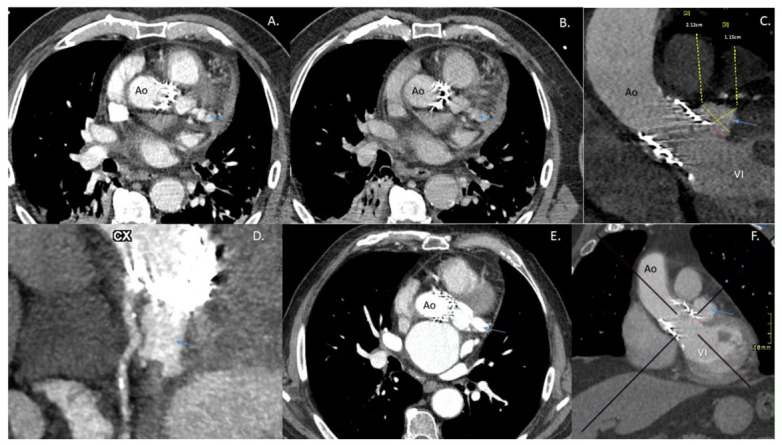
Cardiac CT: (**A**) Pseudoanerysm arterial phase. (**B**) Venous phase confirming pseudoaneurysm. (**C**) Pseudoanerysm 1.5 × 2 cm. (**D**) Pseudoaenurisma below circumflex artery. (**E**) Arterial phase pseudoaneurysm growth. (**F**) Pseudoaneurysm growth (comparison with image (**C**)). Ao: Aorta. CX: Circumflex. VI: Left ventricle. Blue arrow: Pseudoaneurysm. Red line: Neck of pseudoaneurysm.

**Figure 3:**
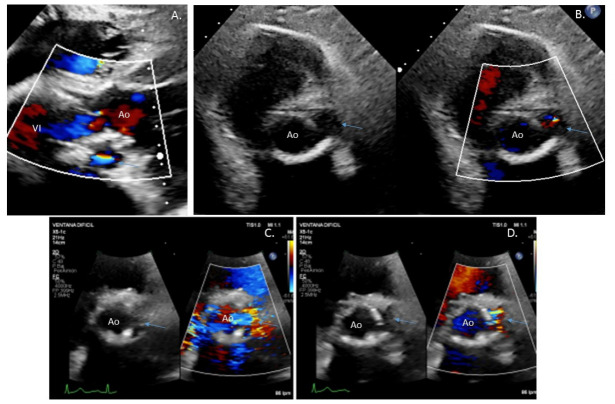
First transthoracic echocardiogram: (**A**) Three-chamber pseudoaneurysm with Doppler; (**B**) short axis pseudoaneurysm with/without Doppler. Second transthoracic echocardiogram without changes compared to the 1st; (**C**) short axis pseudoaneurysm with/without Doppler. (**D**) Absence of shunt/fistula. Ao: Aorta. VI: Left ventricle. Blue arrow: Pseudoaneurysm.

**Figure 4:**
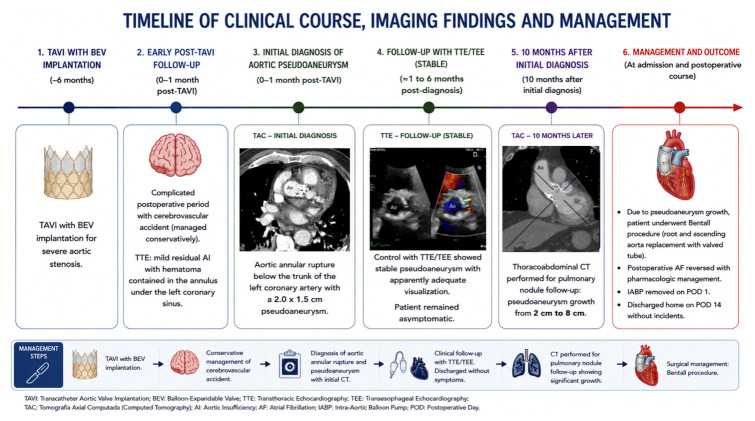
Timeline of clinical course, imaging findings and management. CT: Computed tomography.

**Table 1:** Aortic anulus rupture phenotypes. AAR: Aortic anulus rupture.

Proposed AAR Phenotype	Main Characteristic	Clinical Presentation	Imaging Features	Clinical Course/Management
**Phenotype 1—Free rupture**	Uncontained annular rupture with blood extravasation	Hemodynamic instability, cardiac tamponade, cardiogenic shock	Pericardial effusion/tamponade on TTE/TEE or CT	Surgical emergency requiring immediate intervention
**Phenotype 2—Fistula**	Formation of a communication between cardiac chambers or vascular structures	Left-to-right shunt, may remain asymptomatic or mildly symptomatic	Doppler flow communication identified on TTE/TEE and/or CT	Often benign or stable; management individualized according to shunt severity
**Phenotype 3—Contained rupture**	Localized pseudoaneurysm contained by adjacent structures	Frequently asymptomatic at presentation	Pseudoaneurysm visualized on CT; echocardiography may underestimate progression	Requires serial CT surveillance due to risk of delayed expansion and need for surgical repair

## Data Availability

All relevant data are within the manuscript.

## Abbreviations

AAR	Aortic annulus rupture
AS	Aortic stenosis
BEV	Balloon-expandable valves
CPB	Cardio-pulmonary bypass
CT	Computed tomography
LVOT	Left ventricular outflow tract
IABP	Intra-aortic balloon pump
TAVI	Transcatheter aortic valve implantation
TEE	Transesophageal echocardiogram
TTE	Transthoracic echocardiography
